# Morphology transition of the primary silicon particles in a hypereutectic A390 alloy in high pressure die casting

**DOI:** 10.1038/s41598-017-15223-w

**Published:** 2017-11-03

**Authors:** J. Wang, Z. Guo, J. L. Song, W. X. Hu, J. C. Li, S. M. Xiong

**Affiliations:** 1 School of Materials Science and Engineering, Tsinghua University, Beijing, 100084 China; 2 Key Laboratory for Advanced Materials Processing Technology, Ministry of Education, Beijing, China; 3 R&D Center, China FAW Group Corporation, Changchun, 130011 China

## Abstract

The microstructure of a high-pressure die-cast hypereutectic A390 alloy, including PSPs, pores, α-Al grains and Cu-rich phases, was characterized using synchrotron X-ray tomography, together with SEM, TEM and EBSD. The Cu-rich phases exhibited a net morphology and distributed at the boundaries of the α-Al grains, which in turn surrounded the PSPs. Statistical analysis of the reconstructed 1000 PSPs showed that both equivalent diameter and shape factor of the PSPs exhibited a unimodal distribution with peaks corresponding to 25 μm and 0.78, respectively.) PSPs morphology with multiple twinning were observed and morphological or growth transition of the PSPs from regular octahedral shape (with a shape factor of 0.85 was mainly caused by the constraint of the Cu-rich phases. In particular, the presence of the Cu-rich phases restricted the growth of the α-Al grains, inducing stress on the internal silicon particles, which caused multiple twinning occurrence with higher growth potential and consequently led to growth transitions of the PSPs.

## Introduction

The wear property of the hypereutectic Al-Si alloys could be significantly improved by the primary silicon particles (PSPs)^1–4^. Massive production of the Al-Si engine blocks was mostly achieve by high pressure die casting (HPDC). Because of fast filling and rapid solidification in HPDC, the PSPs exhibit quite different morphologies from these in other casting processes^5–7^.

The morphology of the PSPs could be altered using different approaches, including high current pulsed electron beams^8^, electromagnetic stirring^9^, ultrasonic vibration^10^, squeeze casting^11^, spray deposition^12^, friction stirring^13^, selective laser melting^14^, modification in casting process^15^, and alloying (phosphorus^16–18^ or rare element^19–22^). Taghiabadi *et al*.^1^ showed that an addition of 0.7wt.% Fe increased the hardness and improved the wear resistance of the F332 Al-Si alloy. Bidmeshki *et al*.^20^ found that adding Mn could improve the wear performance of the alloy by changing the morphology of the Fe-rich intermetallic. Shi *et al*.^23^ found that the dominant wear mechanisms for Al–20%Si–0.3%Nd alloy were abrasive wear, adhesive wear and oxidative wear, while for Al–20%Si alloy only abrasive wear and adhesive wear were presented. Raghukiran *et al*.^24^ found that the Al-Si-Sc alloy exhibited significantly high wear performance because both morphology and size of silicon particles were altered by adding Sc. However, to the best knowledge of authors, very limited work has been performed to investigate the Cu effect on the PSPs, e.g. in a A390 alloy which is extensively applied in aerospace and automotive industries.

The synchrotron X-ray image technique makes it possible to characterize the 3-D morphology of the microstructure without damaging the sample, and extensive studies have been performed to investigate dendrites^25,26^, defects^27,28^, PSPs^29^ and other phases^30^.

In this study, we employed synchrotron X-ray tomography to characterize the 3-D morphology of the PSPs in a hypereutectic A390 alloy processed using HPDC. Further analysis *via* SEM, TEM and EBSD was performed on the solidification microstructure with particular attention focused on the interaction between PSPs, α-Al dendrites and Cu-rich phases to disclose the underlying mechanism that controls the morphological transition of the PSPs.

## Experiment

**Figure 1:**
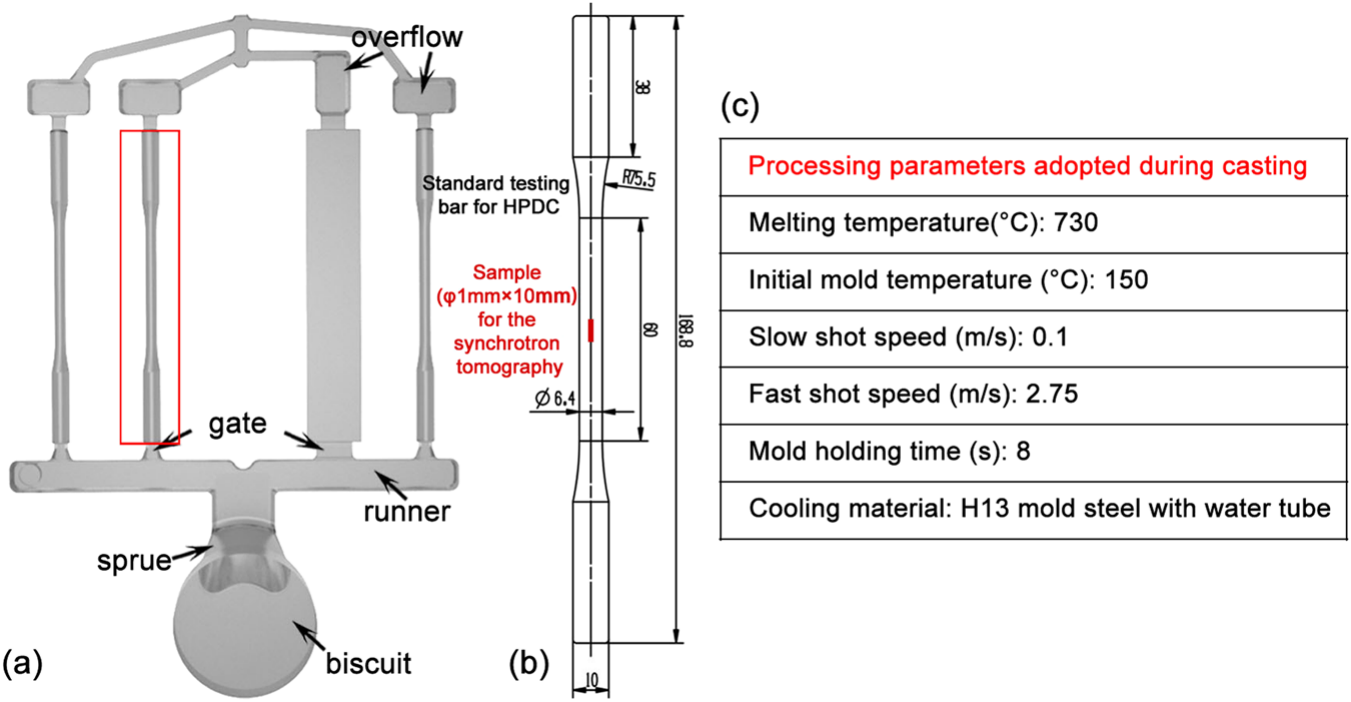
(**a**) Configuration of the HPDC casting, (**b**) location of the specimen for synchrotron X-ray tomography experiment, and (**c**) associated HPDC processing parameters.

In experiment, a commercial hypereutectic Al-Si alloy, namely A390 was employed, nominal composition of which is shown in Table 1. A TOYOBD–350V5 cold chamber die casting machine was applied to produce standard tensile testing bars (ø 6.4 mm) and Fig. 1 shows the sample configuration, together with the processing parameters^27,28^.

**Table 1:** Chemical composition of the A390 alloy.

Type[wt.%]	Si	Cu	Mg	Fe	Zn	Mn
A390	17.10	4.58	0.56	0.32	0.06	0.01

The metallographic samples were extracted from the tensile testing bar (see Fig. 1), mounted in a holder, grinded using SiC papers (400–2000 grit size) and then polished with ethyl alcohol based colloidal silica suspension (0.5 μm). The specimens were then etched by a hot aqueous solution of 20wt.% NaOH at 80 °C for 100 seconds, which helped to reveal the silicon particles by removing the rest microstructure. The 0.5wt.% HF solution was used to remove eutectic and Cu-rich phases so that the PSPs and α-Al dendrites could be clearly rendered. After ultrasonic cleaning in the ethanol solution, the stereo morphologies were observed using a Zeiss Merlin FESEM equipped with energy dispersive X-ray spectrometer (EDX). The polished cylinder was subjected to an electron probe micro-analyzer (EPMA, JXA-8530F) to determine the distribution of Al, Si and Cu. For the EBSD analysis, the specimens were ground with SiC paper, polished for 15 minutes and then cleaned in the ethanol solution with ultrasound. The EBSD experiment was performed using a TESCAN MIRA3 LMH scanning electron microscope with HKL Channel 5 system, which allowed to determine the twin grain boundaries (TGB). Samples for the TEM observation were prepared by mechanical grinding sections from 1 mm to ~100 μm in thickness, and 3 mm in diameter. These samples were then milled by ion beam (Ar^+^) with Gatan 691 ion milling machine after dimpling. Characterization of the grain boundaries (including TGB) of PSPs was performed using TecnaiG^2^ F20 TEM.

The synchrotron X-ray tomography experiment was performed at Shanghai Synchrotron Radiation Facility (SSRF) using the beamline namely BL13W1. A high speed CCD camera was used to record the penetrable intensity of the monochromatic X-ray beam *via* rotating the sample over 180°. The sample, i.e. a ø1 mm cylinder, was retrieved from the casting center. The X-ray energy was set to be 18.5 keV and the distance between the specimen and camera was 20 cm. After scanning, a total of 720 projections of image were collected. Dark field (without X-ray penetration) and flat field images (without samples) were also collected for image processing. The raw 32 bit images were then converted to 16 bit using a software namely PITRE developed by INFN Trieste^31,32^. The 3D reconstruction and rendering of the images were then performed on a sub-volume of 780 × 780 × 1200 voxels with a voxel size of 0.65 μm using the software namely Avizo®^33^.

**Figure 2:**
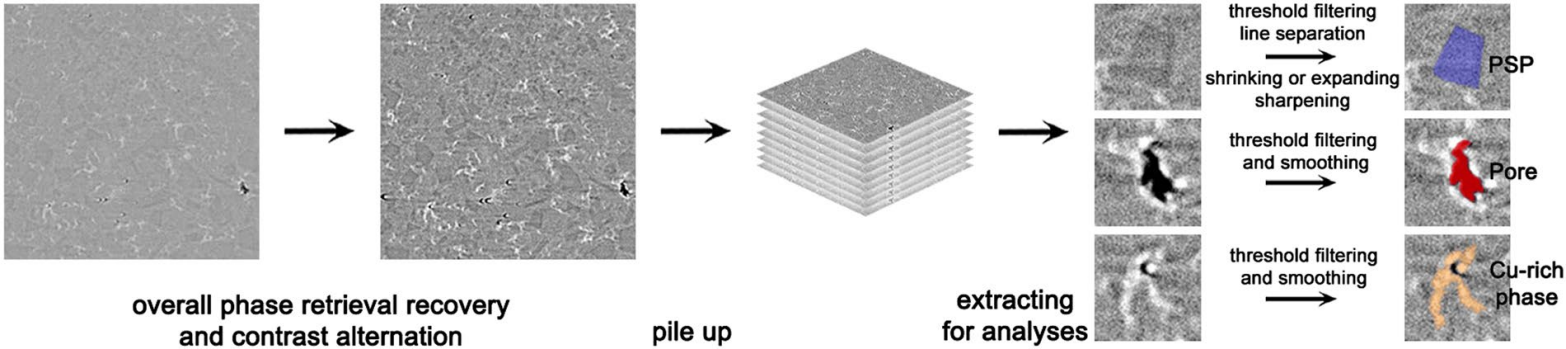
Image processing procedures and methods employed for extracting microstructures.

Image processing techniques such as contrast conditioning, thresholding and smoothing were applied during the data processing^25,26,29^. Figure 2 shows a detailed image processing procedure. After phase retrieval using PITRE, the image contrast was strengthened to distinguish the different microstructure. After converted to 16-bit, the image data was imported into a software namely Avizo® for microstructure extraction. In particular, pores and Cu-rich phases were extracted using threshold filtering and smoothing, while PSPs were extracted using threshold filtering and line separation method. If the extracted area could not be clearly resolved, both shrinking or expanding method was used, together with the sharpening method. The microstructure such as pores, Cu-rich phases and PSPs could thus be clearly identified. The 3-D morphology of the microstructures were then reconstructed based on the processed 2-D images. Further smoothing or sharpening modules were applied to remove the zigzag surfaces for the 3-D morphology.

## Results

### Typical microstructure of A390 alloy

**Figure 3:**
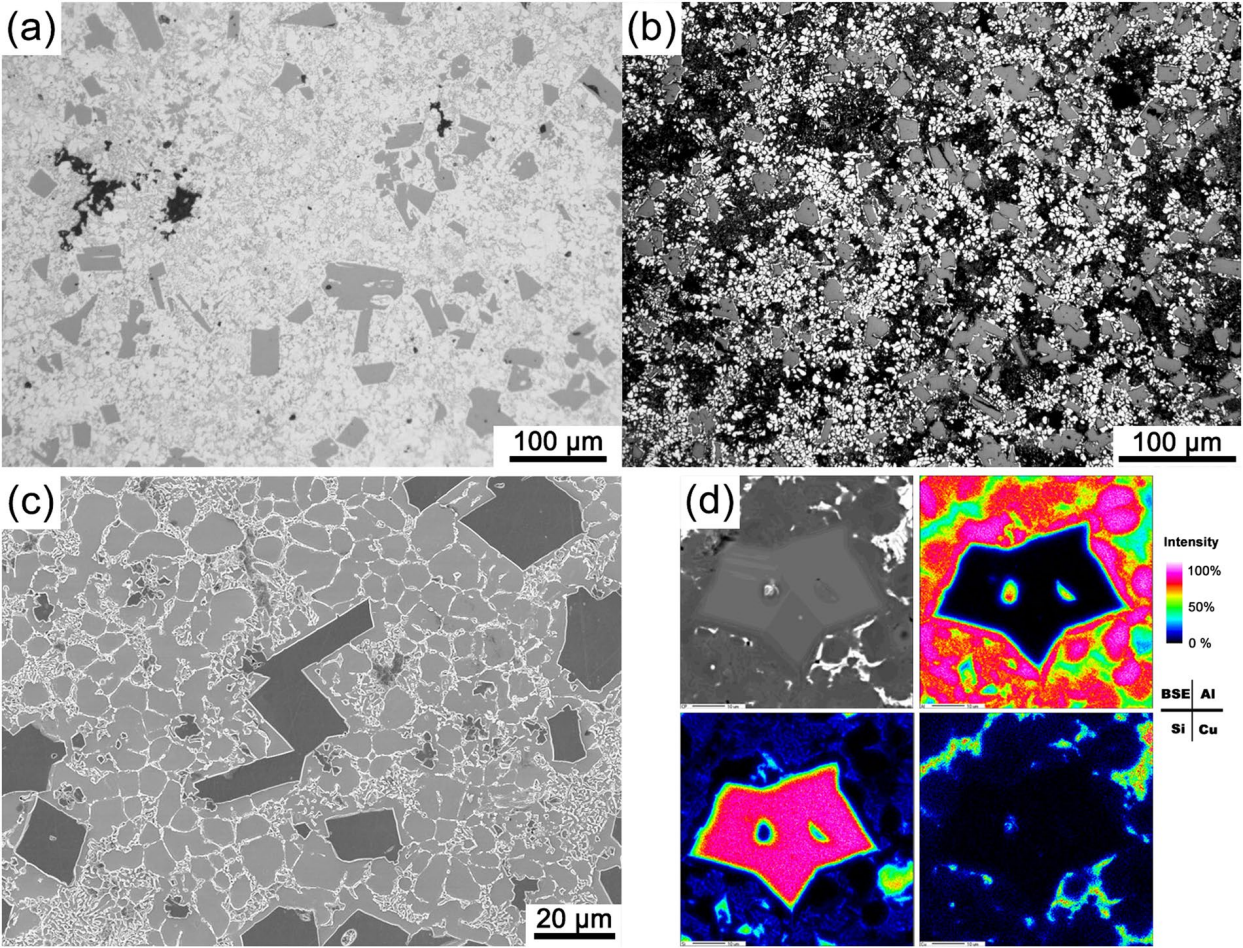
Micrograph of the HPDC A390 alloy microstructure, showing optical micrograph (**a**) before etching and (**b**) after etching, (**c**) SEM image of etched surface and (**d**) EPMA results for the microstructure.

Figure 3a shows a typical microstructure of the HPDC A390 alloy, in which PSPs, α-Al dendrites, Cu-rich phases and pores could be clearly identified. Comparing with the microstructure under slow cooling conditions^29,34,35^, the morphology of the PSPs in HPDC were quite different. The elongated PSPs exhibited tetragons, which were not cross-sections of a typical octahedron or twinned morphology. After etched by a HF solution to remove both eutectic and Cu-rich phases, the microstructure was further observed *via* optical micrograph and SEM. As shown in Fig. 3b and c, the PSPs were closely surrounded by the α-Al dendrites, and most α-Al dendrites exhibited a spherical morphology instead of the commonly observed tree-like shape (or dendrite)^36^. Such morphology of the α-Al dendrite was similar to that of the Al-20%Si alloy, i.e. when Cu element was not present^35^. Figure 3d shows the EPMA result for the Al, Si and Cu elements. The PSPs were surrounded by spherically distributed Al element, i.e. representing the α-Al dendrite. The Cu-rich phases were distributed in a net morphology at the α-Al dendrite boundaries. The EPMA result shown in Fig. 3d revealed that the Cu element or the Cu-rich phase was mainly distributed at the grain boundaries of the α-Al dendrite. The Cu-rich phases, mainly comprising Al_2_Cu, Al_3_CuNi^37^, Al_5_Cu_2_Mg_8_Si_6_, Al_7_Cu_4_Ni and Al_7_(Cu,Ni)_2_Fe^38^, exhibited bright color in both SEM and synchrotron X-ray tomography images.

### Reconstructed microstructure using X-ray tomography

**Figure 4:**
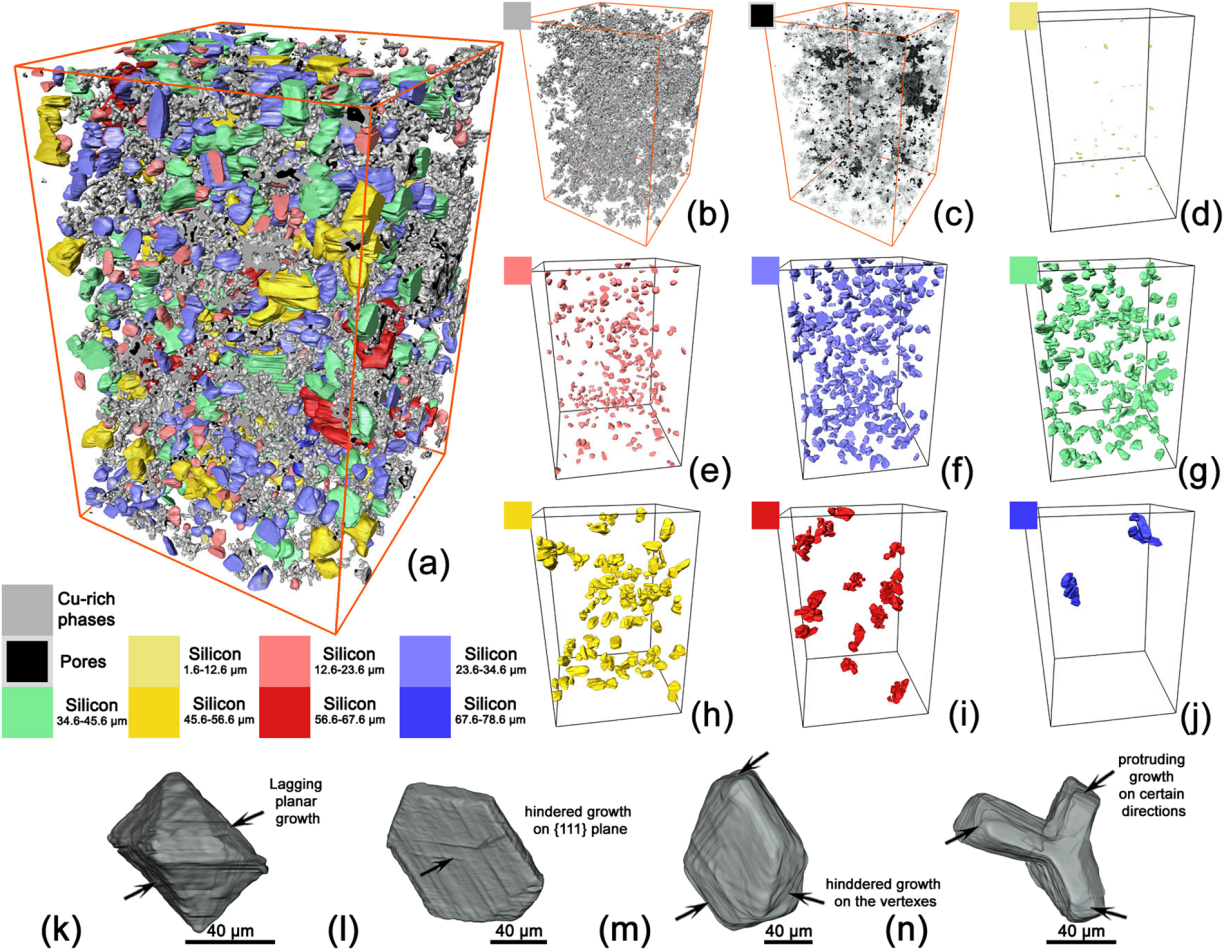
(**a**) Reconstructed microstructures of the A390 alloy, including (**b**) Cu-rich phases, (**c**) porosity and (**d**)–(**j**) PSPs with different size or equivalent diameter. (**k**)–(**n**) show PSPs with atypical or irregular shapes.

Figure 4a showed the reconstructed 3-D morphology of the Cu-rich phases, pores and PSPs in a domain of 507 × 507 × 780 μm^3^, detail of which was shown in Fig. 4b,c and d–j, respectively. As shown in Fig. 4a, the PSPs could be classified into seven categories according to the equivalent diameter. As shown in Fig. 4a and b, the Cu-rich phases exhibited a typical inter-connected net morphology, which was quite different from that of the plate shape Fe-rich intermetallics^38,39^ or Ni aluminides^30^. Most pores tended to locate and distribute inside the Cu-rich phases.

Comparing with other hypereutectic alloys^29,34^, the PSP morphology of the current alloy exhibited much less octahedral or twinned shapes, and asymmetrical or irregular PSPs of 40–80 μm were commonly observed, as shown in Fig. 4k–n. The PSPs in Fig. 4k and l exhibited irregular octahedral shapes, possibly caused by inhibited growth of certain {111} planes. Fig. 4m shows a hexagonal prim shape formed due to the inhibition growth of both planar and vertex directions. Figure 4n shows a “Y” shape PSP formed due to significant growth difference between <111> and <11$\overline{2}$> directions.

### The size and geometry distribution of PSPs

**Figure 5:**
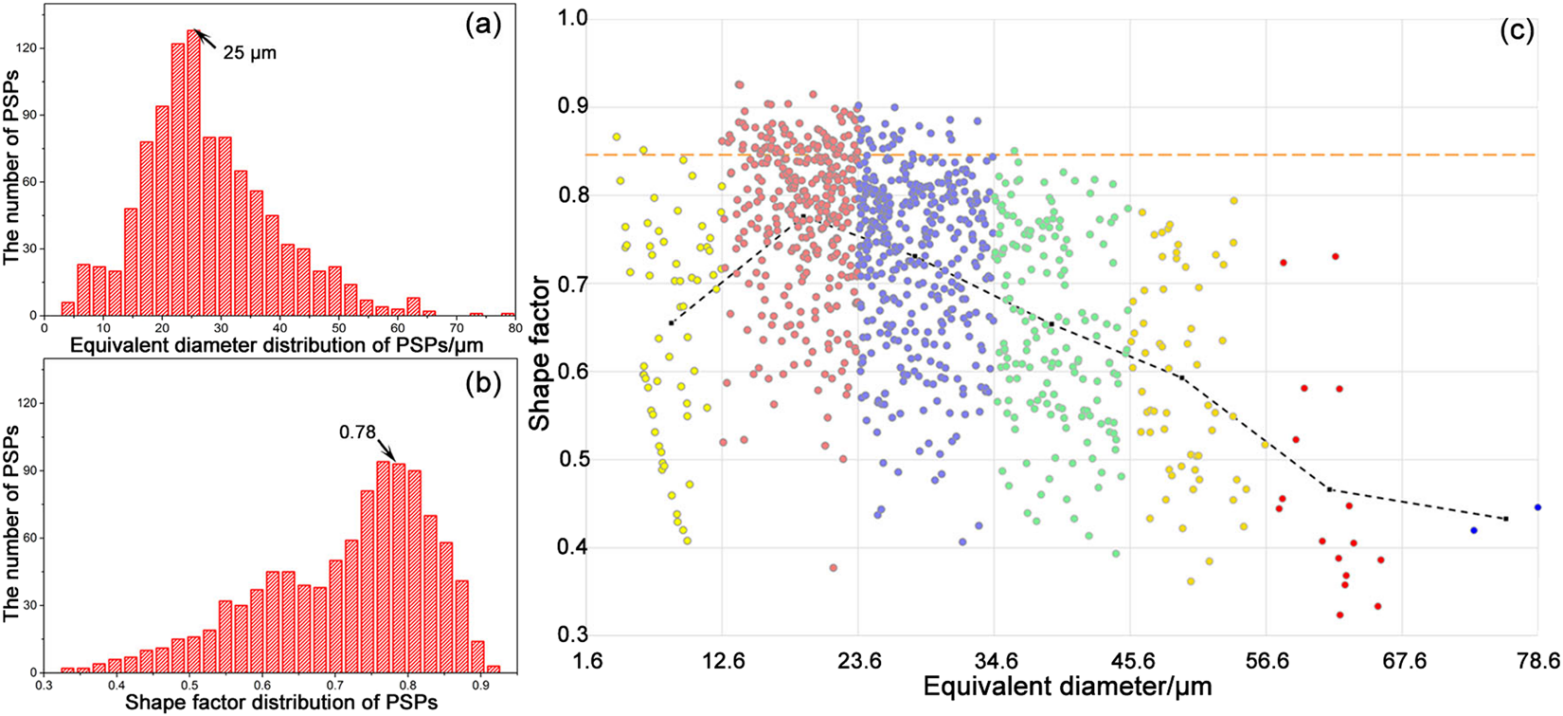
Morphological feature distribution of the PSPs. (**a**) and (**b**) showed the histogram distribution of the equivalent diameter and shape factor of the PSPs, respectively. (**c**) showed the change of shape factor versus the equivalent diameter of PSPs according to the classification in Fig. 4. (The horizontal dashed line marked the theoretical shape factor of PSPs and the bending dashed line was plotted by connecting the average values of shape factor in each size category.)

Here, the equivalent diameter *d*_*equ*_ and shape factor *S*_*F*_^40^ were employed to characterize the morphology of the PSPs:

1 $${d}_{equ}=\sqrt[3]{6V/\pi }$$

2 $${S}_{F}=\sqrt[3]{36\pi {V}^{2}}/A$$

where *V* and *A* are volume and surface area of a particle. Figure 5a and b show the morphology distribution of the PSPs. Both equivalent diameter and shape factor exhibited a unimodal distribution and most PSPs had an equivalent diameter of 25 μm. The most probable PSPs had a shape factor of 0.78, i.e. smaller than that of a regular octahedral shape, which was 0.85 based on its geometry configuration. The deviation of the shape factor from the theoretical value of regular shapes indicated large distortion of the morphology, which was mostly induced by rapid solidification during HPDC.

Figure 5c shows the distribution of the shape factor *versus* equivalent diameter for all PSPs. For the PSPs over 18 μm, the shape factor decreased with the increasing size, as marked by the dash line. Besides, larger PSPs always had lower shape factor, indicating less symmetry, or in other words, the PSPs tended to grow along particular directions. For instance, the two large PSPs (~70 μm) shown in Fig. 4j exhibited clear branches and consequently had lower shape factor. For PSPs of 45 μm–70 μm, half of PSPs had a shape factor of ~0.5, also indicating an irregular growth. On the other hand, smaller size PSPs (4 μm–10 μm) had a shape factor of 0.4–0.9, indicating a rather diverse morphological features.

## Discussion

### Irregular growth of the PSPs

**Figure 6:**
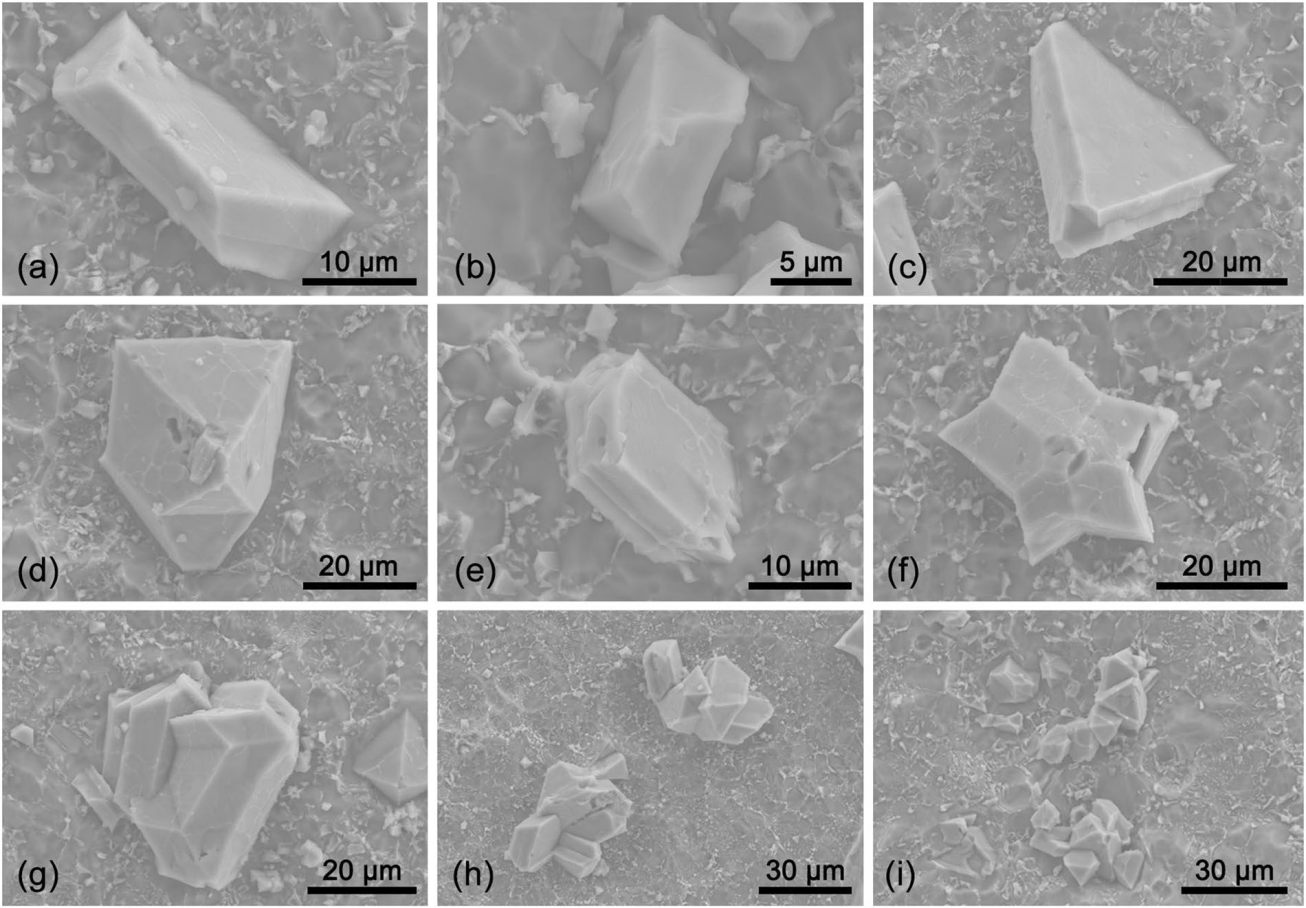
PSPs with irregular morphologies observed *via* SEM.

Figure 6 shows PSPs with irregular shapes, i.e., different from typical octahedral or twinned shapes. Figure 6a and b show plate-like PSPs, formation of which was mostly due to growth inhibition on the two {111} planes. Figure 6c to e show PSPs with similar twinned shape. Beside {111} plane, twinning also occurred near both corner and vertex. Multiple twinning, including parallel twinning (based on the {111} planes) and twinning along central axis, could also be observed, as shown in Fig. 6e and f. The parallel twinning offered parallel re-entrant groove for atom absorption and thus promoted the growth of edges. For the twinning along central axis, Pei and Hosson^41^ employed the twin plane re-entrant edge (TPRE) mechanism to explain radial branching and growth along <110> . They found that tiny small-angle grain boundaries (SAGBs) exhibited because the total twinning angle was 352.5° (70.5° × 5). In Fig. 6g–i, clustering PSPs was observed and exhibited multi-time twinning. This explained the existence of large PSPs with low shape factor as shown in Fig. 4h–j. The growth of smaller PSPs was easily constrained by the {111} planes and a facetted morphology, e.g. plate-like shape was formed. On the other hand, for larger PSPs twinning offered new growth directions when PSPs were constrained, which consequently led to branch-like (see Fig. 6h) or chain-like morphology (see Fig. 6i).

**Figure 7:**
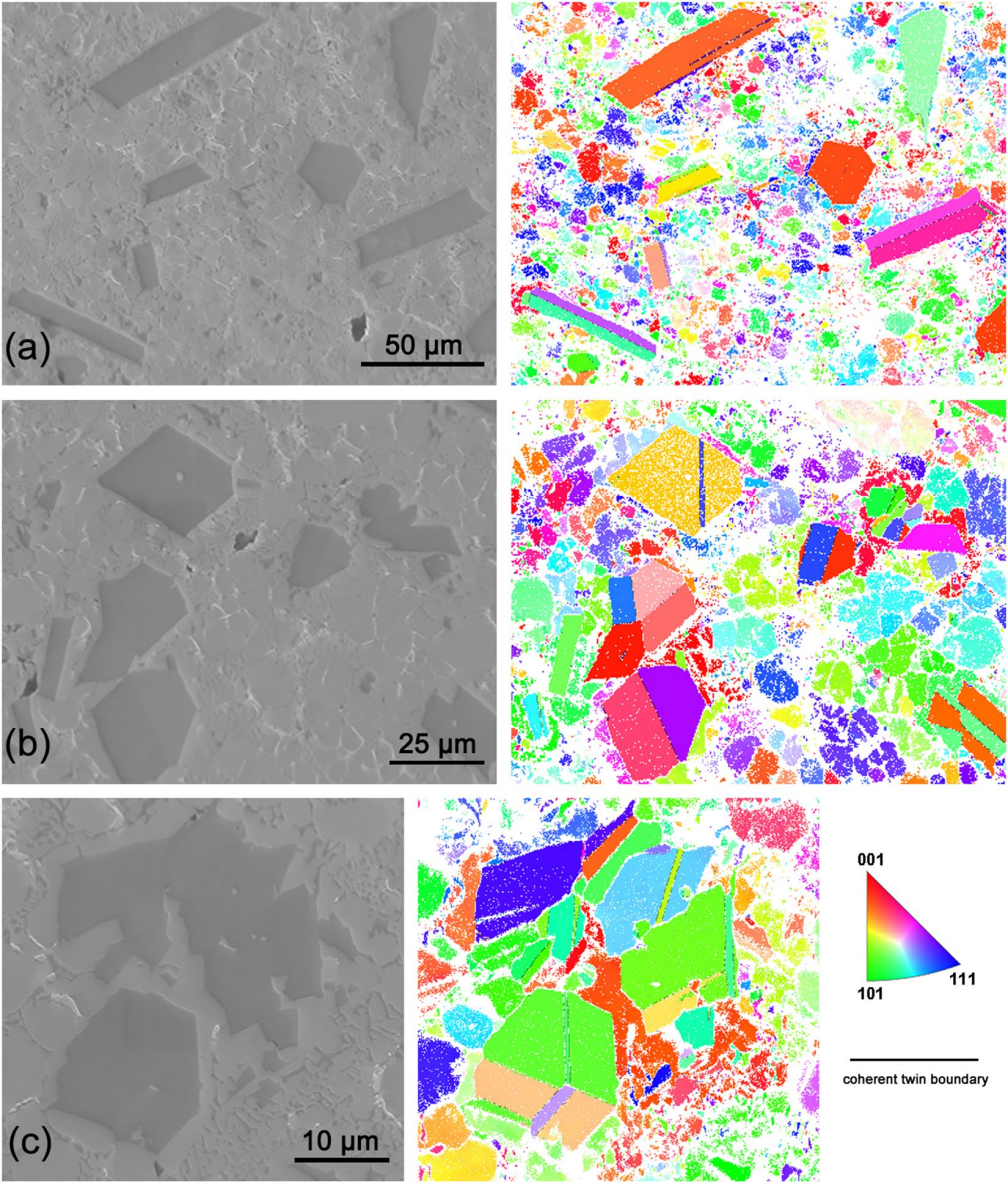
EBSD results of PSPs with multiple twinning occurrences.

Figure 7 shows the EBSD result on the distribution of crystal orientation and high density coherent twin boundary of the PSPs. Figure 7a shows thin layer twinning at the center or edge of the plate-like PSPs. Figure 7b and c show other polyhedral shape PSPs with thin layer twinning. Multiple twinning could also be observed for some octahedral shape PSPs. As one of the main growth mechanisms of PSPs^40^, twinning could decrease the surface energy but increase the bulk strain energy.

Table 2 shows the measured contact area between PSPs (pores) and the Cu-rich phases. The contact area percentage was the contact area between PSPs (pores) and Cu-rich phases divided by the overall surface area of the PSPs (pores). As shown in Table 2, the contact area between PSPs and Cu-rich phases increased with the number of PSPs but the contact area percentage remained at ~1%, indicating that the Cu-rich phases had no direct effect on the size or morphology transition of PSPs. The morphology of PSPs was quite different from that when the additional element was P^17^, Nd^42^, Ce^36^ or Er^43^. For the latter cases, the growth of PSPs was restricted and twinning was induced because the solute atoms worked as obstacles at the solid-liquid interface. The pinning effect of solute atoms retarded the silicon growth and altered the particle morphology. As shown in Fig. 3, both eutectic and Cu-rich phases were distributed in-between neighboring α-Al dendrites instead of contacting the PSPs. Because of indirect contact, the influence of Cu element on the PSP morphology was different from that of P, Nd, Ce or Er.

**Table 2:** Measured contact surface area (percentage) between PSPs (pores) with Cu-rich phases.

Microstructure	Surface area/μm^2^	Contact area with Cu-rich phases/μm^2^	Contact area percentage/%
PSPs between 1.6–12.6 μm	24408.2	290.1	1.2
PSPs between 12.6–23.6 μm	502254.7	4819.3	1.0
PSPs between 23.6–34.6 μm	1340190.2	15365.3	1.1
PSPs between 34.6–45.6 μm	1300223.4	10215.1	0.8
PSPs between 45.6–56.6 μm	839682.7	9016.6	1.1
PSPs between 56.6–67.6 μm	441952.3	7806.4	1.8
PSPs between 67.6–78.6 μm	83882.8	793.3	0.9
Pores	569665.8	405778.5	71.2

### Solidification path with rapid cooling rate

The specimen was extracted in the center of the bar to avoid the surface chilling effect. According to the thermal analysis in^37^, the growth of PSPs during solidification comprised three stages: large-scale silicon agglomeration, nucleation and growth of the PSPs. In silicon, the diffusion coefficient of Cu was ~10^−8^ m^2^/s (~600 °C), which was much higher than that of Al, indicating during silicon agglomeration, Cu diffused much faster than Al. As solidification, both Al and Cu elements was rejected in the melt, promoting the nucleation and growth of PSPs. Comparing with Cu, Al diffused much slower and tended to remain at the boundary of PSPs, which consequently led to the formation of α-Al dendrites. The Cu element on the other hand would remain at the external boundary of the α-Al dendrites. The spheroidisation of the α-Al dendrites was caused by dendritic fragmentation and coarsening^44–46^, and the presence of the Cu-rich phases restricted the microstructure for further growth, like α-Al dendrites and PSPs.

### The driving force for twinning

Al has a *fcc* lattice structure with *a* = 4.0490 Å while for Si, it is diamond with *a* = 5.4282 Å^47^. The growth mode for the α-Al dendrite was cubic with preferred growth directions like <100> but for silicon, it was layered based on the close-packed {111} planes^40,48,49^. Accordingly, the absorption of Al atoms to promote α-Al dendrite growth was more efficient than that of Si for PSPs. Further growth of the α-Al dendrites hindered the growth of PSPs by isolating Si atoms. At the phase boundary between α-Al dendrite and PSP, the lattice mismatch caused the formation of thin-stacking-layer Al-Si eutectics and twinning, in particular layer-like twinning based on {111} planes^50^. As restricted by the surrounding microstructure, twin boundaries of PSPs like Σ3, Σ9 and Σ27 could be identified in the EBSD map. The twinning could also induce higher order parasitic twinning or parallel twinning based on {111} planes. The morphology of PSPs, either reconstructed *via* synchrotron X-ray tomography or etched *via* SEM, showed parallel {111} planes with polyhedron shape. The silicon facets promoted nucleation of twins when they were located at the edge of the sample or at grain boundary grooves^51^.

**Figure 8:**
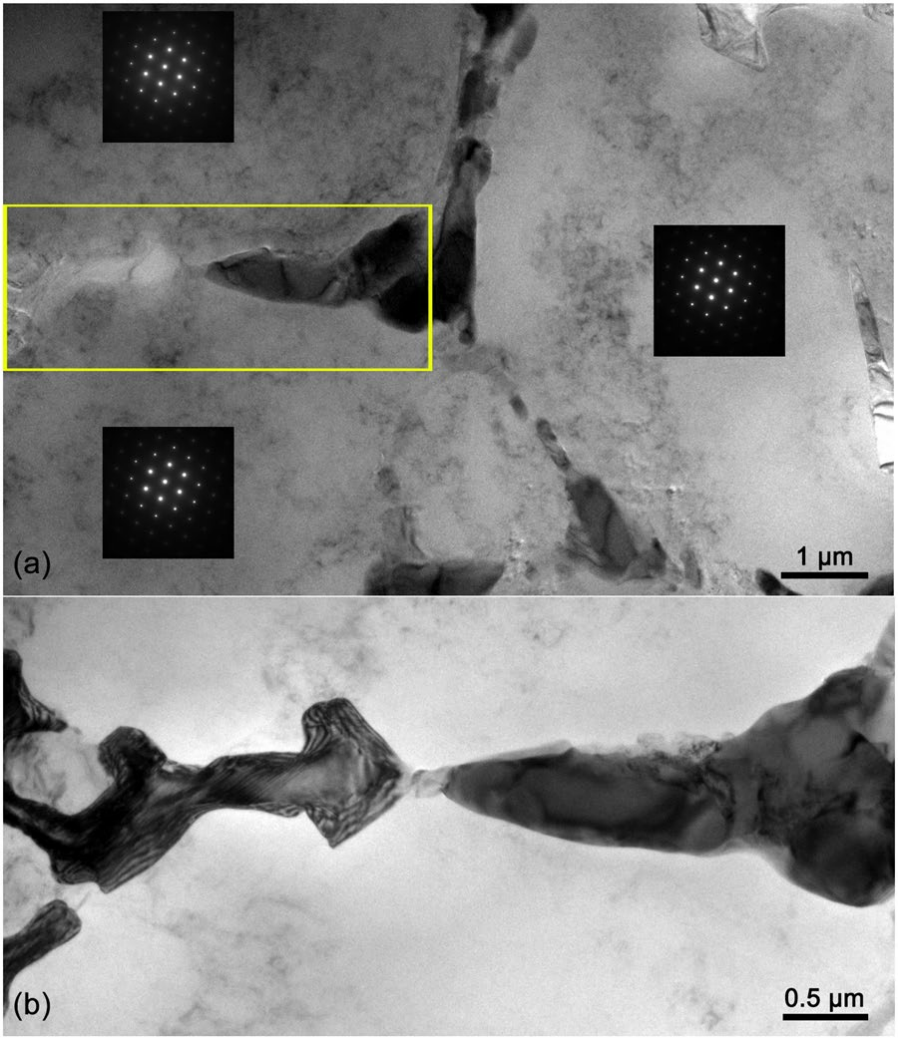
TEM images of Cu-rich phases and the α-Al dendrites. (**a**) shows three diffraction patterns of α-Al dendrite parts. (**b**) shows the successive Cu-rich phases as marked by the rectangle in (**a**).

Figure 8 shows the TEM images of Cu-rich phases and the α-Al dendrites. The inter-connected Cu-rich phases were normally located at the boundaries of the α-Al dendrite, which was similar to the situation for the A319 alloy^52^. Further growth of either α-Al dendrites or PSPs was constrained by the interconnected Cu-rich phases. Accordingly, the PSPs tended to change the growth direction *via* twinning on the {111} plane, or constrained completely with a limited size.

**Figure 9:**
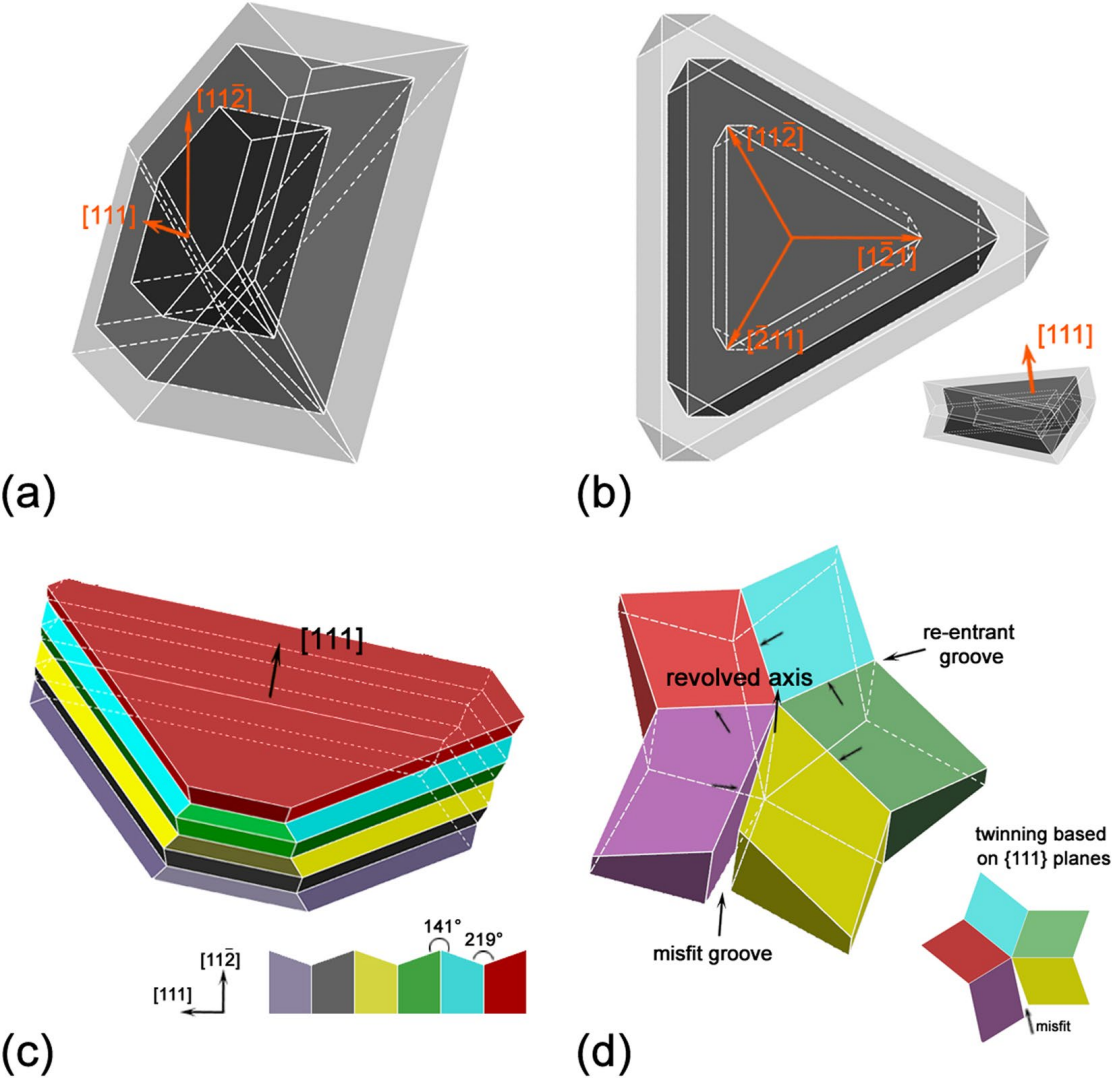
Schematic illustration for the growth mechanism of twinning of PSPs.

Figure 9 shows a schematic illustration of the irregular growth of PSPs. The growth of the octahedral shape was mainly achieved *via* atom absorption according to the diamond cubic structure. However, such growth was inhibited due to lack of Si atoms caused by the presence of aluminum dendrites. In this respect, it was difficult for PSPs to grow symmetrically along <111> directions in an octahedral shape but rather in a plate-like shape because certain {111} planes were hindered, as shown in the Fig. 9a. However, if twinning occurred, the growth of PSPs was promoted due to the enhanced growth ability along the three <11$\overline{2}$> directions by increasing possible the re-entrant grooves. In Fig. 9b, the typical twinning occurred and the three equivalent <11$\overline{2}$> directions (or six when the opposite directions were included) tripled the growth chance with three re-entrant edges. The alternation growth of direction helped further growth of PSP which consequently formed into a plate-like morphology. Besides, enough inhibition on the {111} plane would cause the occurrence of twinning, especially multiple parallel twinning, and the re-entrant grooves increased. In this respect, the growth potential along the three <11$\overline{2}$> directions was enhanced with increasing possibility to absorb silicon atom in the grooves, comparing with that on an atomically flat {111} plane^40^. The twinning might occurred in such way that twinning planes were based on the revolved axis. Accordingly, possible growth directions for PSPs increased because of multiple twinning though defects would exist in the PSP due to assembling misfit of different twinning parts.

As the growth along <111> was inhibited, twinning offered ways *via* plate-like expansion, such as the triangular prism mechanism in Fig. 6c when three twinning directions grew in a similar speed, or the hexagonal prism mechanism in Fig. 4l when six twinning directions grew similarly. The twinning also offered ways to create more steps or “defects” to lower the total free energy of the system *via* two multiple twining mechanisms. As shown in Figs 6e and 9c, during twinning, the {111} planes piled up one by one, creating more steps with parallel re-entrant grooves^53^. Twinning could also take place when the five {111} planes revolved over certain axis, achieving a star-like morphology with a 7.5° misfit angle^41^. Further growth of this shape proceeded by absorbing atoms at the five re-entrant grooves and/or even the defect grooves^54^. Other types of twinning also took place on certain {111} planes and the PSPs would exhibited crystal-like shapes as shown in Fig. 6g–i.

### Evidence of microstructure stress

**Figure 10:**
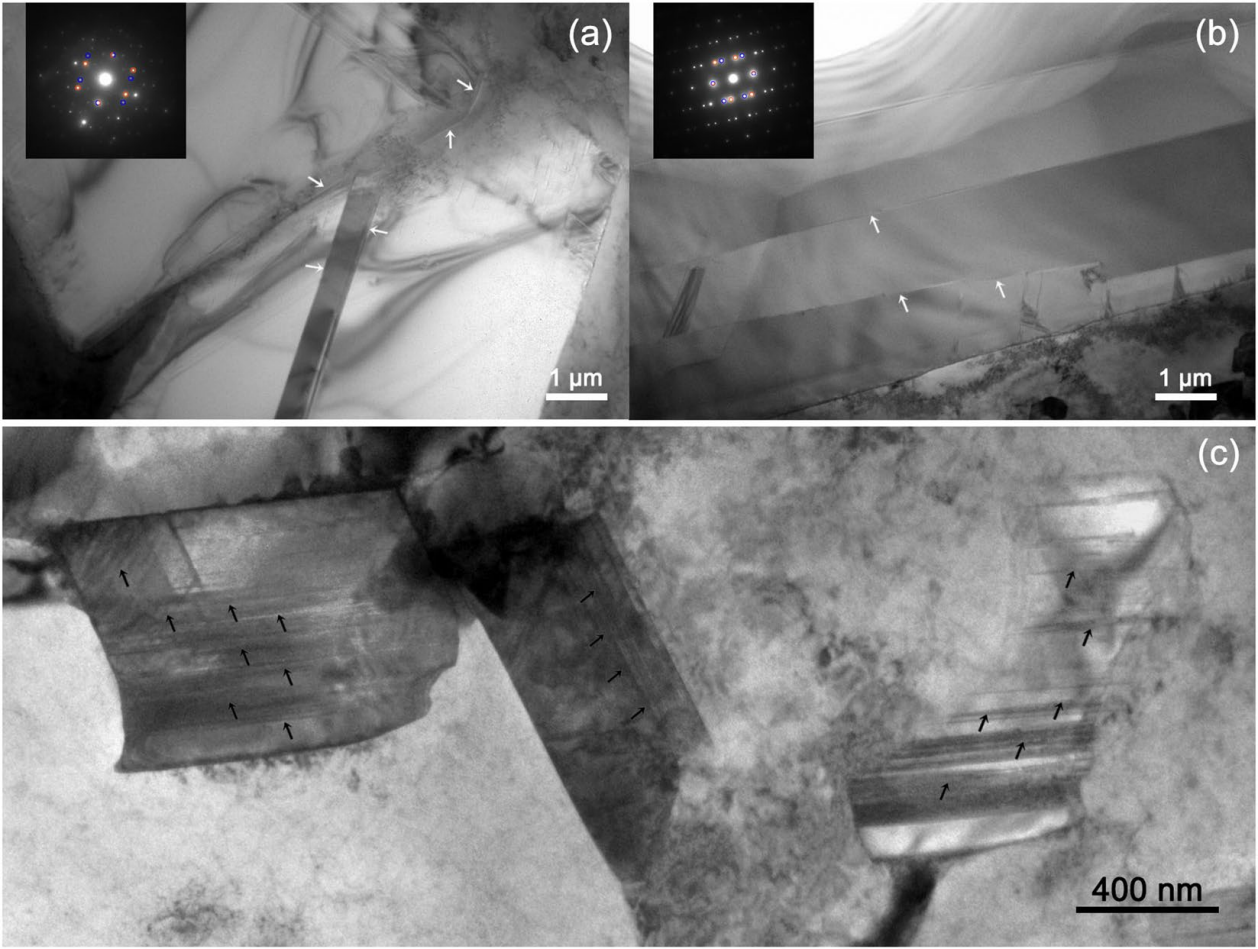
TEM images for twinning (marked with arrows) during the growth of one PSP in (**a**) and (**b**); TEM images for stacking faults (marked with arrows) in the PSPs in (**c**).

Figure 10a and b show the intersecting plane twinning and multiple layer twinning. Figure 10c shows high-density stacking faults in PSPs of size ~1 μm. According to the EPMA and TEM observation, inside the PSP was almost pure Si and few other element exhibited, indicating a zero influence of the heterogeneous element on the stacking fault. The stress, on the other hand, could be the most probable reason for the stacking faults. If the stress was caused by macro external forces, deformation defects could also be induced for ductile microstructure like α-Al dendrites. However, further microstructure analysis indicated that this is not the case. In this respect, the stress must be local and induced by surrounding microstructure, e.g. the squeezing from α-Al dendrites, which was also observed in^55^. Comparing with the Al-20wt.%Si alloys^29,34^, the additional squeezing effect from the α-Al dendrites on the PSPs could be induced by the inclusion of Cu in the alloy. The net-like Cu-rich phases constrained the further growth of both α-Al dendrites and the PSPs.

## Conclusion

In this work, the microstructure of a high-pressure die-cast hypereutectic A390 alloy was characterized using synchrotron X-ray tomography, together with SEM, TEM and EBSD. Particular attention was focused on the morphology and distribution of PSPs, pores and Cu-rich phases inside the solidification microstructure. Accordingly, the following conclusions can be drawn:

1. The microstructure of the A390 alloy in HPDC comprised PSPs, pores, α-Al dendrites and net-like Cu-rich phases. For PSPs, both equivalent diameter and shape factor exhibited a unimodal distribution and most PSPs had an equivalent diameter of 25 μm and shape factor of 0.78. The net-like Cu-rich phases distributed uniformly in the microstructure at the grain boundaries of the α-Al dendrites.
2. Comparing with typical octahedral and twinned shapes, the morphology of the PSPs in the current A390 alloy changed significantly. Statistical analysis revealed that most PSPs exhibited a shape factor of *S*_*F*_ = 0.78, which was different from that of a regular octahedral shape, i.e., *S*_*F*_ = 0.85. The morphology transition of the PSPs was mainly attributed to twinning (multiple and/or parallel).
3. The net-like Cu-rich phases restricted the growth of α-Al dendrites, which further constrained and caused morphological transition of the PSPs. High density stacking faults exhibited in PSPs (<1 μm) but for α-Al dendrites no deformation defect was observed. The restriction of the Cu-rich phases on PSPs was believed to be the most significant reason for the stacking fault.
